# Case report: Complications after using the “blind-stitch” method in a dairy cow with a left displaced abomasum: treatment, outcome, and economic evaluation

**DOI:** 10.3389/fvets.2024.1470190

**Published:** 2024-11-12

**Authors:** Melanie Schären-Bannert, Lilli Bittner-Schwerda, Fanny Rachidi, Alexander Starke

**Affiliations:** Clinic for Ruminants and Swine, Faculty of Veterinary Medicine, University of Leipzig, Leipzig, Germany

**Keywords:** transition cow, cattle, left displaced abomasum, abomasopexy, omentopexy, togglepin

## Abstract

A fourth lactation dairy cow that was 35 days in milk was referred to the clinic for treatment after undergoing unsuccessful treatment of a left displaced abomasum (LDA). The physical examination revealed complications after using the “blind-stitch” method for correction of the LDA; the cow had an abnormal general demeanor, decreased gastrointestinal motility, and local inflammation of the abdominal wall at the site of the suture. Systemic antibiotics, anti-inflammatory drugs, and intravenous fluids were administered, and a right flank laparotomy and omentopexy according to Dirksen were performed after cutting the suture and breaking down the adhesions of the localized peritonitis between the abdominal wall and the abomasal puncture site. The cow was monitored clinically and discharged 2 weeks after referral. The cow was milked for another two lactations producing a total of 18,000 kg of milk, with a lifetime production of 59,141 kg. The total cost for the case was 310 € for the first intervention using the “blind-stitch” method and 897 € for the second laparotomic intervention. The costs (excluding tax) of both procedures including physical examination, surgery, medications, diagnostics, and labor were calculated. The lost revenue associated with the withdrawal period and lower milk production was 4,168 €. Percutaneous LDA fixation techniques, such as the “blind-stitch” and “toggle-pin” methods, have gained popularity because they are quick and cost-effective and involve minimal labor. However, many buiatricians are critical of these techniques because of the high risk of complications. The following four factors require careful consideration when choosing a method for LDA correction: (1) Percutaneous methods require precision and adherence to the described inclusion, exclusion, and cancelation criteria; (2) Operator skill is essential, and therefore regularly performing laparotomies increases surgical experience and enables the veterinarian to better manage different and more complex abdominal disorders; (3) By performing a laparotomy, other underlying abdominal disorders such as reticuloperitonitis and abomasal ulcers may be detected; (4) Postoperative husbandry and treatment are important factors affecting the outcome. The cost calculated for this case underlines the potential benefit and necessity of successful animal health management and the importance of a highly skilled veterinarian and farm workforce.

## 1 Introduction

Left displaced abomasum (LDA) is a common disease of transition dairy cows (1). It is multifactorial in nature, and breed, ration, housing management, and other factors play a role (1–3). It is often associated with other transition cow diseases, has a severe impact on production and longevity, and has economic as well as animal welfare implications (1). The herd-level incidence of LDA in modern dairy production systems ranged from 2.5 to 4.8% in one study (1) and 0.05 to 5.8% in another (4). Thus, the most frequently performed abdominal surgery in dairy cows is the correction of LDA (4–7).

Over the years, different medical and surgical methods for correction of LDA have been developed and evaluated (7–20). Surgical methods include laparotomy as well as laparoscopic and percutaneous techniques such as “blind-stitch/tack” or “toggle-pin fixation (5, 6, 21–24). The so-called “toggle-pin” technique has become popular because it is quick, may be carried out by laypeople, and is generally considered cost-effective (6, 25, 26). The “toggle-pin” technique has a distinct advantage over the “blind-stitch” method because when the abomasum is punctured, the odor of abomasal gas confirms accurate placement of the toggle (27). Some studies found no difference between the “toggle-pin” technique and laparotomy with regard to milk production and survival of cows (28, 29), but another study showed that percutaneous methods have a high complication rate (19). These methods are therefore viewed critically by many buiatricians because they involve the risk of accidentally puncturing other organs and structures instead of the greater curvature of the abomasum as well as accidentally inducing a rupture into the abomasum (5, 19, 22, 24, 27, 30).

The present case report aims to highlight the controversy regarding the surgical methods for LDA treatment by describing the diagnosis, treatment, and outcome of a cow referred to our clinic with complications associated with the “blind-stitch” method for correction of LDA. In addition, the economic implications are outlined and some aspects of the choice of different abomasal fixation techniques, which in our opinion have not been explained sufficiently, are discussed.

## 2 Case description

### 2.1 Case history

A 4th lactation dairy cow, 35 days in milk (DIM), was referred to the Clinic for Ruminants and Swine, University of Leipzig, because of a poor response to “blind-stitch” fixation of LDA (timeline in Table 1). The cow originated from a 500-cow dairy herd and was transported more than 104 km to our clinic, which took approximately 2.5 h. The herd manager reported that the cow had a current milk production of 16 kg per day and had undergone the “blind-stitch” method for correction of LDA 2 days earlier. The cow had been treated in the 10 previous days with dexamethasone (three consecutive days 15 mL of Dexamethason-Injektionslösung ad us. vet. 2 mg/mL, Serumwerk Bernburg AG, Bernburg, Germany) and menbutone (50 mL on 5 of 10 days of 100 mg/mL, Menbutil, aniMedica GmbH, Senden-Bösensell, Germany), ketoprofen (20 mL on two of 10 days of Romefen^®^ PR 10%, Ceva Tiergesundheit GmbH, Düsseldorf, Germany), calcium (100 mL of Calcitat S50, Livisto/ animedica GmbH, Senden-Bösensell, Germany), oral propylene glycol (250 mL on 6 of 10 days, product label unknown), phosphorous and cyanocobalamin (30 mL on 5 of 10 days of Veyxol B-Phos, Veyx-Pharma GmbH, Schwarenborn, Germany), and rumen stimulans (300 g on 5 of 10 days of Pansenreaktiv, alfavet Tierarzneimittel GmbH, Neumünster).

**Table 1:** Time table of the case.

Timepoint	Event
1st lactation	calving at 23 months of age, milk production of 12,764 kg (3.92% fat, 3.53% protein, 451 days in milk (DIM), 39 d dry)
2nd lactation	milk production of 12,190 kg (4.25% fat, 3.41% protein, 328 DIM, 68 d dry)
3rd lactation	milk production of 14,927 kg (4.52% fat, 3.58% protein, 390 DIM, 45 d dry)
4th lactation	diagnosis of left displaced abomasum (LDA) in 4^th^-5^th^ week of lactation and treatment with “blind-stitch” method, dexamethasone, butaphosphan, and cyanocobalamin
day 0	arrival at clinic at 35 DIM, clinical examination reveals acute severe complication of unsuccessful “blind-stitch” fixation with a LDA, acute moderate inflammation of the fixation site, and severe acute localized peritonitis. Treatment with antibiotics and non-steriodal anti-inflammatory drugs, and infusion therapy.
day 1	laparatomy, detachment of abomasum from adhesions, suturing of abomasal lesion site and omentopexy
day 2–14	continuing antibiotic and non-steroidal anti-inflammatory therapy until day 10, infusion therapy until day 7, administration of drench on day 1, intraperitoneal administration of antiseptic solution of day 1 and 3. Gradual improvement of health status.
day 3 and 13	ultrasound examination of abdomen showing gradual reduction of local peritonitis lesion
day 14	discharge from clinic
day 290	finishing the 4^th^ lactation with 7,885 kg of milk (4.67% fat, 3.59% protein, 325 DIM, 32 d dry)
5^th^ lactation	milk production of 10,291 kg (4.64, 3.63% protein, 332 DIM, 89 d dry)
6^th^ lactation	euthanasia at 28 DIM due to downer cow syndrome after severe abduction of the hind-legs.

### 2.2 Clinical findings

**Figure 1:**
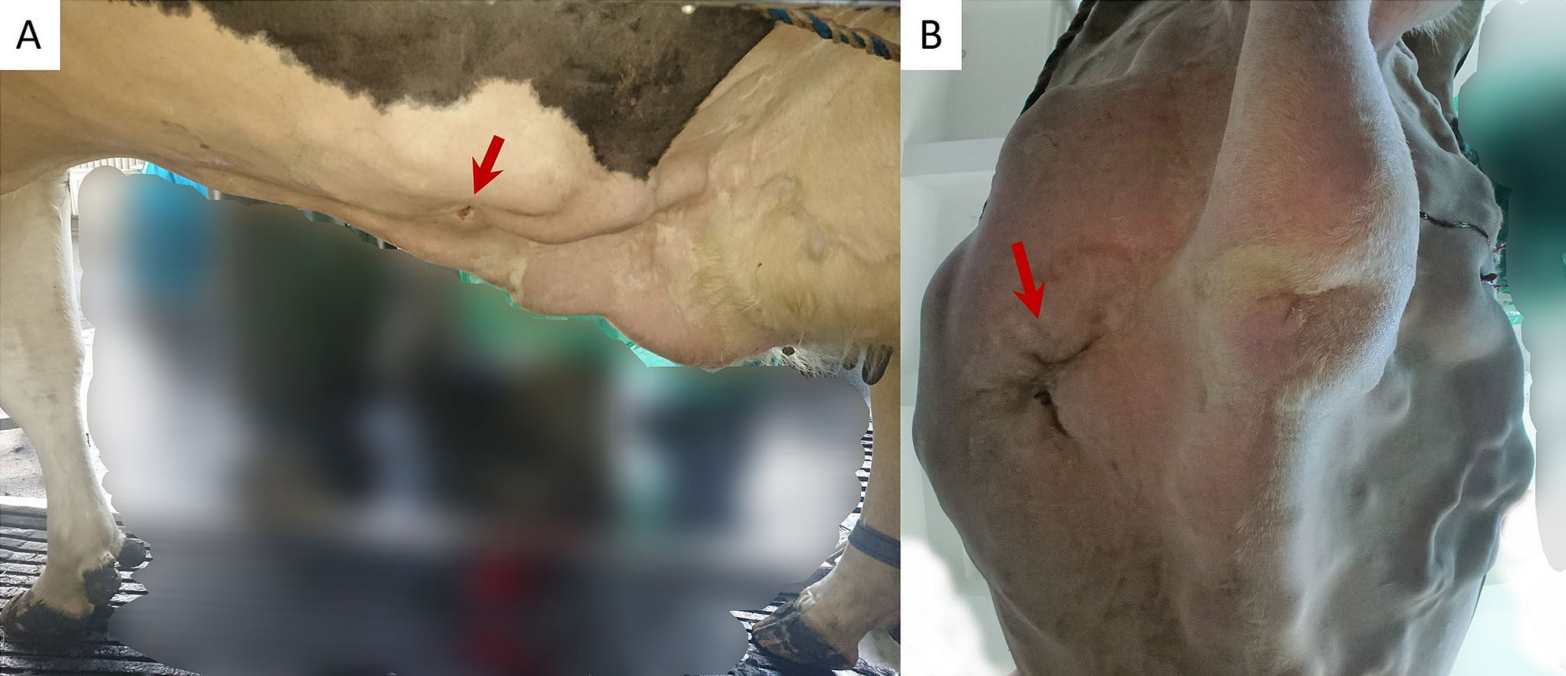
Photograph of the patient from the left **(A)** and below **(B)** showing the suture (arrow) of the “blind-stitch” method, surrounded by severe edema in the left ventral area of the abdomen, approximately 5 cm cranial to the navel and 10 cm paramedian.

Clinical examination revealed that the cow was acutely ill and had a slightly depressed demeanor and a body condition score of 2.25 (31). The vital signs were unremarkable; the rectal temperature was 38.9°C, the respiratory rate was 30 breaths per min, and the heart rate was 64 beats per min. Auscultation of the lungs and heart revealed no abnormal findings. The eyes were mildly sunken, and the skin turgor was slightly decreased. Rumen motility was absent, and rumen fill was poor, and stratification was absent. A ping sound could not be elicited on the right side of the abdomen but was prominent on the left, accompanied by splashing sounds over a wide area of the rib-supported part of the abdominal wall up to the height of the scapula. The density of the right abdominal wall was slightly increased. The manure was olive green in color and of medium consistency with some undigested long fibers. Suture material was palpable in the left paramedian area of the abdomen, approximately 5 cm cranial to the navel and 10 cm from the midline. The suture was in the center of a painful edematous swelling that was approximately 20 × 20 cm (Figure 1). Based on the severely abnormal demeanor of the cow, absent gastrointestinal motility, and moderate localized inflammation at the suture site, an acute severe complication of unsuccessful “blind-stitch” fixation was suspected.

Collection of a blood sample from a jugular vein for hematologic and biochemical analyses and a free-flow urine sample was done immediately after the physical examination (Table 2). The results showed an acute inflammatory process and tissue damage based on a high total leukocyte count and increased serum aspartate aminotransferase. In addition, decreases in dry matter intake and intestinal passage rate were reflected by lower than normal concentrations of serum magnesium, potassium, and urea, higher than normal serum bilirubin concentration, and a low urine chloride concentration (32). Ultrasonography revealed that the abomasum was located to the left of the median and extended dorsally between the rumen and abdominal wall. Echogenic structures indicative of fibrinous deposits were seen around the suture material, wound, and abomasum within the abdominal cavity (33).

**Table 2:** The results of hematologic and serum biochemistry analyses in a cow with complications attributable to “blind-stitch” correction of LDA.

Trait^1^	Unit	Reference^2^		Admission		One day post-OP	
Hemogram							
Leukocytes	10^9^/L	5–10		11.0		12.4	
Erythrocytes	T/L	5–10		7.41		6.76	
Hemoglobin	mmol/L	5.5–8.1		7.3		6.8	
Hematocrit	L/L	0.24–0.46		0.33		0.29	
MCV	fl	45–65		45.1		42.3	
MCH	fmol	0.9–1.5		1.0		1.01	
MCHC	mmol/L	16–21		21.9		23.9	
Thrombocytes	10^9^/L	100–600		242		412	
Hemogram Differentiation^3^							
		%	10^9^/L	%	10^9^/L	%	10^9^/L
Band neutrophils		24–42	1.3–4.5	–	–	82	10.17
Lymphocytes		45–65	2.5–6.5	–	–	17	2.11
Monocytes		2–9	0.1–0.9	–	–	1	0.12
Minerals / Electrolytes / Trace Elements							
Mg	mmol/L	0.90–1.32		0.69		0.72	
Ca	mmol/L	2.00–2.54		2.40		2.04	
P	mmol/L	1.55–2.29		1.62		1.44	
Na	mmol/L	135–157		146		143	
K	mmol/L	3.9–5.2		3.64		3.98	
Cl	mmol/L	95–110		101		108	
Protein / Metabolism							
TP	g/L	68–82		73.2		60.5	
Alb	g/L	30–39		34.4		28.3	
Bili	μmol/L	(3.3)-5.3		9.0		5.7	
Urea	mmol/L	2.0–6.8		1.82		2.23	
Crea	μmol/L	55–150		112		112	
GT^4^	min	> 15 min		> 15 min		-	
Enzymes							
AST	U/L	< 80		106		164	
GGT	U/L	< 50		35.2		31.7	
GLDH	U/L	5–30		6.9		48.6	
CK	U/L	< 200		153		1,548	
Urine							
Cl	mmol/L	40–160		16		–	

1 Alb, albumin; AST, aspartate aminotransferase; Bili, bilirubin (total); Ca, calcium; CK, creatine kinase; Cl, chloride; Crea, creatinine; GGT, gamma-glutamyl transferase; GLDH, glutamate dehydrogenase; K, potassium; MCH, mean corpuscular hemoglobin; MCHC, mean corpuscular hemoglobin concentration; MCV, mean corpuscular volume; Mg, magnesium; Na, sodium; P, phosphorus; TP, total protein.

2 Reference values of the Laboratory of Large Animal Clinics, Faculty of Veterinary Medicine, University of Leipzig, chosen according to (32).

3 direct microscopically, no automated count; therefore, no value on the day of admission measured (late afternoon–laboratory closed).

4 GT, Glutaraldehyde Test, reference according to (60).

Left displaced abomasum, acute moderate inflammation of the fixation site, and severe acute localized peritonitis were diagnosed. The fixation site was thought to be at an unsuitable location other than the intendent part of the abomasum.

### 2.3 Treatment

After the initial clinical examination, the cow received an intravenous infusion of 5 L isotonic saline solution and 500 mL 40% glucose (both Serumwerk Bernburg AG). The cow was premedicated with an antibiotic (60 mL Trimethosel; 200 mg/mL sulfamidine and 40 mg/mL trimethoprim, administered intravenously; Selectavet, Dr. Otto Fischer GmbH, München, Germany) and a non-steroidal anti-inflammatory drug (15 mL Metacam; 20 mg/mL meloxicam, Boehringer Ingelheim Vetmedica GmbH, Ingelheim am Rhein, Germany). Distal paravertebral anesthesia and infiltration of the incision site with 200 mL isocaine (20 mg/mL procaine hydrochloride and 0.025 mg/mL epinephrine, Selectavet) were done. Right flank laparotomy was carried out to assess the extent of inflammation and the position of the abomasum and to perform an omentopexy (34).

**Figure 2:**
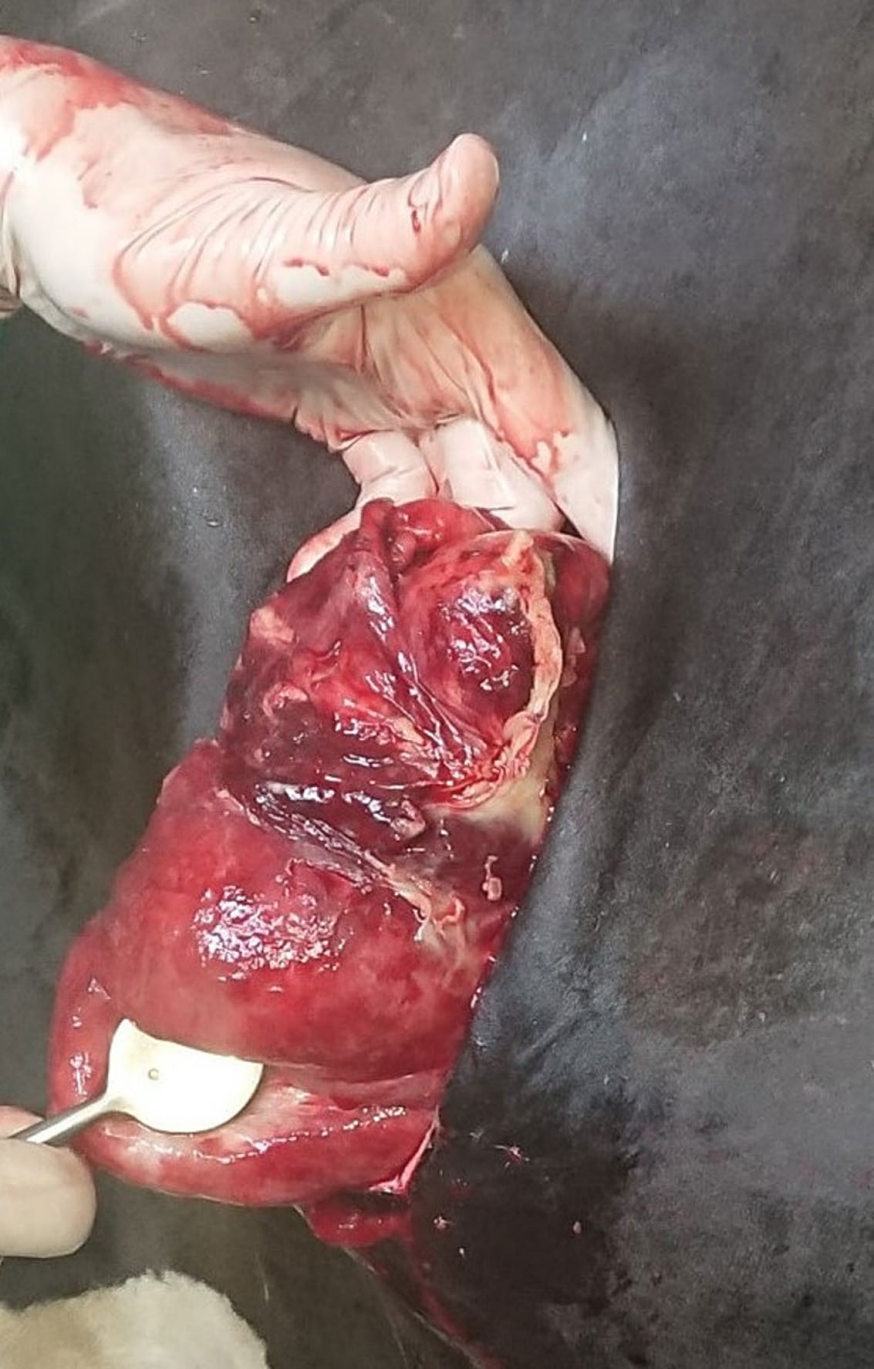
Photograph taken during right flank laparotomy showing localized inflammation and gelatinous and highly perfused tissue in the pyloric area before omentopexy was carried out.

The external skin surrounding the “blind-stitch” suture was cleaned and disinfected to facilitate subsequent removal of the suture material. The laparotomy showed that the pyloric part of the abomasum, approximately 10 cm cranial to the pylorus, was fixed to the ventral abdominal wall. The abomasum was displaced to the left but only slightly distended. The pexy was characterized by severe local fibrinous peritonitis corresponding to an extra-abdominal site of fixation. The liver was enlarged with blunted borders, indicating moderate to severe fatty infiltration attributable to lipomobilization (35, 36). The adhesions were carefully broken down, and the “blind-stitch” suture was cut externally and removed under careful visual monitoring to prevent further injury to the abomasum. The abomasum was then moved to its normal position, palpated, and inspected for ruptures or other lesions (Figure 2). It was cleaned using a 0.5% povidone-iodine solution (diluted Vet-Sept Lösung 10%, aniMedica GmbH, Senden-Bösensell, Germany). The lesion was repaired using inverted sutures, and an omentopexy was carried out (34). A total of 3.5 L of 0.5% povidone-iodine solution was placed in the peritoneal cavity before the incision was routinely closed.

The cow received infusion therapy consisting of 1.5 L of 40% glucose and 20 L of isotonic saline solution on the day of surgery and then approximately 1.5 L of 40% glucose and 10 L of isotonic saline solution per day for 3 days and 1 L of 40% glucose and 5 L of isotonic saline solution per day for the following 4 days. The cow showed signs of pain immediately after surgery and therefore received 50 mL of metamizole-sodium (500 mg/mL, Metapyrin, Serumwerk Bernburg AG) administered intramuscularly. On day 1 postoperatively, 30 L of warm water containing 250 mL propylene glycol and glycerin (Tirsana, H. Wilhelm Schaumann GmbH, Pinneberg, Germany), 250 g cooked linseed, 180 g NaCl, and 3 L of rumen fluid from a fistulated cow at the clinic were administered as a drench. On day 2 postoperatively, 60 mL sulfadimidine and trimethoprim (Trimethosel) was administered intravenously and continued once daily for 10 days. The cow also received 15 mL of meloxicam every other day for 10 days. An antiseptic solution (Vet-Sept) was administered intraperitoneally on day 1 (1 L) and on day 3 (2 L) postoperatively. Daily treatments with 250 mL propylene glycol and glycerin (Tirsana) were continued for 10 days.

The health of the cow improved gradually after surgery. The results of hematologic and serum biochemical analyses 1 day after surgery reflected localized inflammation and tissue damage; the results were similar to those on the day of admission but with a left shift and increased creatine kinase activity (Table 2). Decreased total protein and albumin concentrations and increased glutamate dehydrogenase activity confirmed hepatic injury. The normokalaemia indicated a resolution of the abomasal reflux. The rectal temperature was normal. An ultrasound examination done 3 days postoperatively showed that the peritoneal lesion was approximately 5 × 5 cm with a small amount of free hypoechogenic fluid in the xyphoid region. A second scan done 13 days postoperatively showed a reduction in the size of the inflammatory lesion with no free fluid in the abdomen and normal bi-phasic reticular contractions. The cow was discharged from the clinic 2 weeks after admission.

### 2.4 Outcome

#### 2.4.1 Patient follow-up

The cow finished her 4^th^ lactation with 7,885 kg of milk (4.67% fat, 3.59% protein, 325 DIM, 32 d dry), and in the following lactation produced 10,291 kg (4.64, 3.63% protein, 332 DIM, 89 d dry). However, the cow did not return to her previous production level (1st lactation: 12,764 kg [3.92% fat, 3.53% protein, calving at 23 months of age, 451 DIM, 39 d dry], 2nd lactation: 12,190 kg [4.25% fat, 3.41% protein, 328 DIM, 68 d dry], 3rd lactation: 14,927 kg [4.52% fat, 3.58% protein, 390 DIM, 45 d dry]). At 204 and 224 DIM in the 4 and 5th lactation, the cow was part of routine herd scoring by university clinic assistants. At these time points, the body condition score was 2.5 and 2.75, respectively, and the cow was not lame, and had only minor bald areas and minimal swelling of the hocks. The herd management software (HerdePlus, dsp-Agrosoft GmbH, Ketzin, Germany) showed a single episode of mastitis in the 4th lactation 3 days after discharge from our clinic. In the early part of the 5th lactation, the cow underwent ketosis prevention and had one mastitis event at 18 DIM. During the remainder of the lactation, there were 4 lameness events recorded (horn fissure, white line disease, sole ulcer, digital dermatitis). In the 6th lactation, the cow had one ketosis event at 26 DIM and was euthanized 2 days later because of downer cow syndrome after severe abduction of the hind legs. The lifetime production of the cow was 59,141 kg (4.40% fat, 3.53% protein).

#### 2.4.2 Economic evaluation of the case

The cost of the original LDA surgery and the follow-up medical treatments (first intervention) including lost revenues for this case were estimated. The cost for the treatment on the farm was obtained from the farm account. The working hours invested were estimated from actual on-farm measurements of time needed to perform these types of treatments (37). Labor costs for the herd manager were set at 25 €/h based on our data and experience.

In our cost model, the second intervention including the surgery and medical follow-up treatment was calculated as having been done on the farm to create a more realistic comparison with the situation in practice. The cost included the laparotomy, medications, placement of an ear vein catheter, the initial reassessment and 3 follow-up examinations, 2 hematologic analyses, 1 ultrasound examination, and labor costs for the herd manager. The work done by the veterinarians was priced using the official fee schedule for veterinarians in Germany (38). Drugs were priced according to their recommended retail cost (39).

The milk loss during the 15-d hospitalization period was 132 kg (source: clinic records). Before referral, the milk could not be shipped for 6 d because of a withdrawal period; this amounted to 96 kg based on a daily production of 16 kg on the day of admission plus another 36 kg based on a daily production of 12 kg in our clinic for the 3 days withdrawal period after discharge. The withdrawal periods were set according to the label-use of the drugs administered. Estimation of the potential milk production in the 4 and 5th lactations was based on the production in the 3^rd^ lactation because population data show only minor differences among lactations 3 to 5 (40). The potential loss amounted to 11,678 kg (7,042 + 4,636 kg). The milk price originated from the monthly economical operating branch analysis for 1 year for this specific farm (34.90 €/100 kg energy-corrected milk). Of note, we did not consider a cost reduction for a lower dry matter intake because of decreased milk production, in contrast to another study (39) because we assumed that the cow had increased energy and nutrient requirements during recovery from surgery. This would have made the calculation of DMI difficult. The final calculation resulted in the following amounts:

Costs (examination, surgery, medication, diagnostics, labor; excluding value-added tax):

First treatment and intervention (on farm): 310 €.

Second intervention (in clinic, calculated as on farm): 897 €.

Lost revenues:

Milk loss due to withdrawal periods: 92 €.

Lower milk production: 4,076 €.

Total: 5,357 €.

#### 2.4.3 Farm follow-up

The risks of the “blind-stitch” method and the benefits of other LDA surgical techniques were discussed with the herd manager and the herd veterinarian (4, 8, 22). We emphasized that right-flank laparotomy was the method of choice in a case like this for the following two reasons: (1) the local veterinarian mainly responsible for this farm was a relatively inexperienced surgeon, and therefore routinely operating LDAs would provide surgical experience and confidence in performing other surgical procedures including right displaced abomasum, cecal torsion, and cesarean section; (2) a laparotomy allows the surgeon to explore the abdominal cavity to rule out other commonly encountered conditions, such as traumatic reticuloperitonitis, fatty liver syndrome, abomasal ulcers, and other sequelae of inflammatory processes.

We also provided the herd veterinarian with hands-on surgical instructions at our clinic and helped with the creation of a suitable on-farm treatment and surgical area. The herd manager decided to refer cows with LDA to our clinic until his veterinarian had gained sufficient experience doing laparotomies because several cows had already been culled after the “blind-stitch” method. In the 2 years after this case, the herd manager sent 25 cows with a history of metabolic and/or abdominal disorders to our clinic for treatment. Of 20 cows undergoing LDA surgery, only one died because of severe fatty liver syndrome. The remaining five cases included a cow with indigestion and bronchopneumonia, two cows with abomasal ulcers (1 died), and two cows with right displaced abomasum (1 died). Increasing the herd manager’s awareness of the importance of detecting and operating cows with LDA promptly improved the prognosis considerably, similar to another report (41). Within 2 years of the present case, a facility for medical and surgical treatments was built at the farm, and the herd veterinarian was trained and became proficient at doing laparotomies.

Left displaced abomasum is the “tip of the iceberg” in multifactorial transition cow diseases (42) and therefore on-farm risk analysis and prevention need to be instituted using a holistic approach (43). A system analysis (44) was carried out and identified several deficiencies in housing, management, and feeding as risk factors for transition cow metabolic disorders on this farm. This led to the development of preventive herd health strategies, including better management of feeding and close-up diet, prevention of overcrowding in the fresh-cow pen, and an increase in the intensity of fresh-cow assessments.

## 3 Discussion

This case report describes the consequences of the unsuccessful treatment of LDA using the “blind-stitch” method. A second surgery and aggressive medical treatment allowed the cow to return to the herd, complete 2 lactations, and produce 2 calves. This outcome is in agreement with another report and emphasizes that cows can recover from complications after LDA surgery and return to production provided that professional veterinary care is provided (45).

Operator skill and appropriate postoperative medical management of the patient are critical factors in the success of surgical procedures (46–48). Unfortunately, the choices for correction of LDA have been only partially addressed and not often in depth in the current veterinary literature (4, 10, 21, 22, 49, 50). Percutaneous fixation techniques such as the “blind-stitch” and “toggle-pin” methods are viewed skeptically by many bovine surgeons because of a relatively high occurrence of complications in the field (19, 22, 24, 30). In our experience, complications are more likely to occur when several simple but important criteria, referred to as inclusion and exclusion criteria—determining whether a patient is by principle eligible for a procedure, and cancelation criteria—determining under which circumstances the procedure should not be continued but aborted, are neglected. For instance, percutaneous procedures should be limited to cases in which a distinct ping sound can be heard in the right paramedian region of the abdominal wall (21, 27). Percutaneous techniques should not be used after 5 months of pregnancy (51), and the suture should be cut if the health status and demeanor of the cow deteriorate in the first 48 h or other complications are observed (27, 28, 45). As mentioned in the introduction, the “toggle-pin” technique has the distinct advantage over the “blind-stitch” method that when the abomasum is punctured, the odor of abomasal gas confirms accurate placement of the toggle (27). However, in a controlled prospective study involving experienced bovine veterinarians in a clinical setting, von Freital (19) observed a complication rate of 10.6% (5.8% no fixation possible and 4.8% recurrence of LDA after fixation, *n* = 104) using the percutaneous fixation technique described by Grymer and Sterner (52). In contrast, no complications were encountered in 104 cows with LDA treated with omentopexy as described by Dirksen (34). In a study by Heimberg (27), percutaneous fixation of the abomasum (51) in a similar setting found that cows had a faster recovery and higher feed intake and milk production compared with cows treated with omentopexy as described by Dirksen (34). These results show that surgeon experience plays an essential role in the success rate of a procedure (46) and highlight the importance of good clinical education and surgical competency as day-one skills (53, 54).

When discussing these surgical techniques, it is important to mention that routinely performing right flank laparotomy for correction of LDA is a prerequisite for treating more involved cases (22, 27) and for detecting other abdominal disease processes such as reticuloperitonitis (21). Diagnosis of an atypical or chronically displaced abomasum, for example, when the abomasum is positioned ventrally between the rumen and abdominal wall without a characteristic ping sound, can be difficult and may only be confirmed during an exploratory laparotomy (41). In our experience, clinicians who are proficient in laparotomy techniques rarely use the percutaneous methods, although their use is still widespread. When the inclusion, exclusion, and cancelation criteria are ignored, the complication rate is usually high. Therefore, some herds have adopted a direct-to-slaughter strategy, which negatively affects animal welfare.

The present case report represents several problems that are commonly encountered in dairy cattle medicine. Clinical shortcomings range from failure to detect illness, definitively diagnose the disorder, and institute appropriate treatment and husbandry measures. This led to enormous financial losses and negatively impacted animal welfare (41, 55, 56). Therefore, thorough clinical education of veterinary students, the establishment of protocols for on-farm disease detection and treatment strategies, including surgical methods, and the creation of suitable treatment areas are of major importance (41, 46, 53, 57).

The cost calculated in this case report highlights the enormous economic benefit of sound animal health management. In most relevant dairy cow production diseases, treatment costs represent only a fraction of the expenses and lost revenue attributable to milk withdrawal, decreased milk production, and culling (55, 58, 59). When the cost of this case is multiplied by the number of cows that were culled because of misdiagnosis and inadequate treatment, it becomes clear that a large amount of revenue would theoretically be available for preventive herd health measures.

## Data Availability

The original contributions presented in the study are included in the article/Supplementary material, further inquiries can be directed to the corresponding author.
