# Supplementary figures and images for: Case report: Complications after using the “blind-stitch” method in a dairy cow with a left displaced abomasum: treatment, outcome, and economic evaluation
Source: Front Vet Sci. 2024 Nov 12;11:1470190. doi: 10.3389/fvets.2024.1470190 (PMC11588720; doi:10.3389/fvets.2024.1470190)

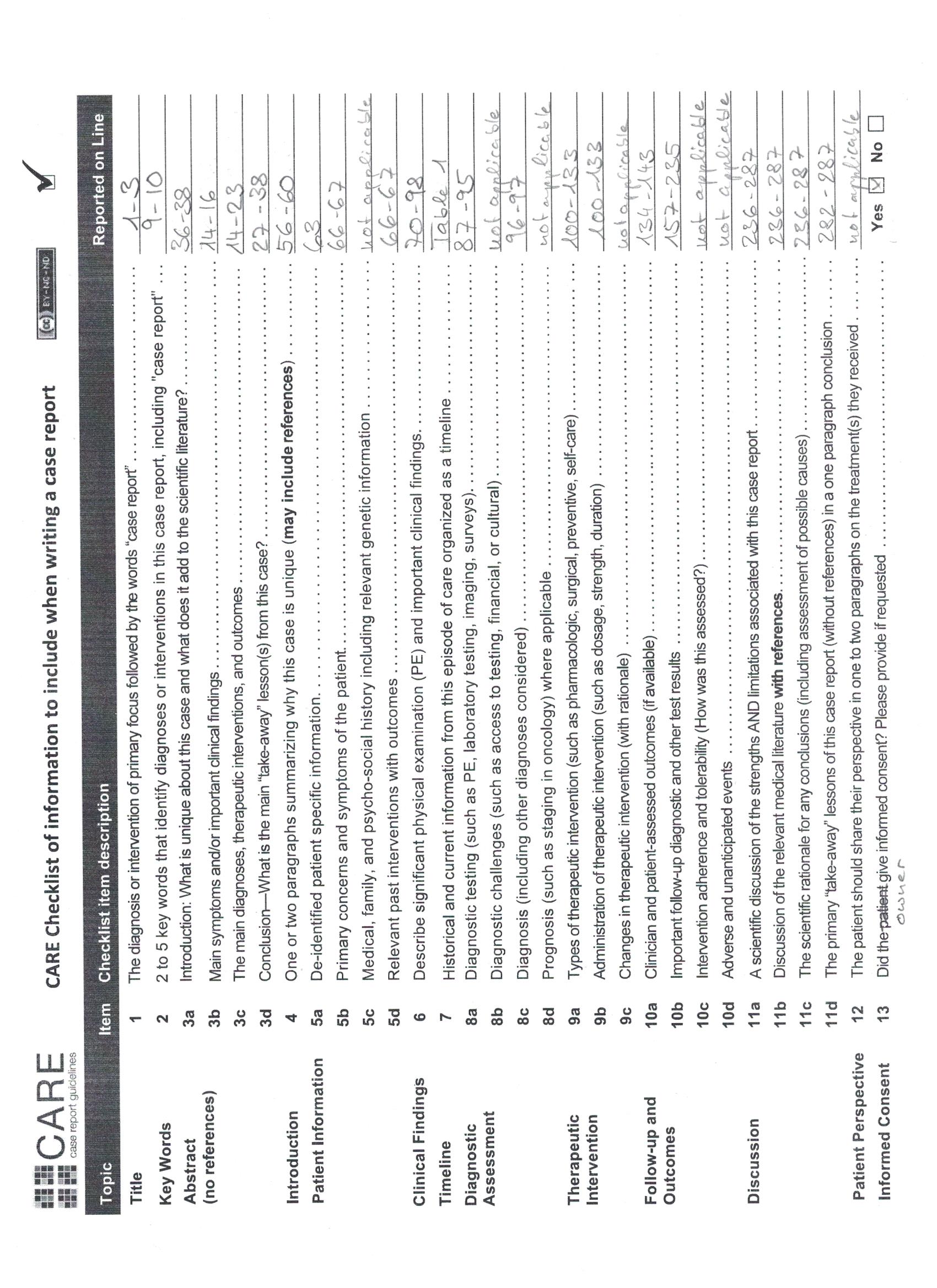

Supplement: Supplementary file 1 [file Image_1.JPEG]
